# Effects of Red Light Night Break Treatment on Growth and Flowering of Tomato Plants

**DOI:** 10.3389/fpls.2016.00527

**Published:** 2016-04-22

**Authors:** Kai Cao, Lirong Cui, Lin Ye, Xiaoting Zhou, Encai Bao, Hailiang Zhao, Zhirong Zou

**Affiliations:** ^1^State Key Laboratory of Crop Stress Biology for Arid Areas, College of Horticulture, Northwest A&F UniversityYangling, China; ^2^Key Laboratory of Protected Horticultural Engineering in Northwest, Ministry of AgricultureYangling, China; ^3^Agriculture College, Ningxia UniversityYinchuan, China

**Keywords:** flowering, fruit fresh weight, hormone, night break, stem elongation, tomato

## Abstract

Compact and healthy young plants increase crop production and improve vegetable quality. Adverse climatic conditions and shading can cause young plants to become elongated and spindly. We investigated the effects of night break (NB) treatments on tomato plants using red light (RL) with an intensity of 20 μmol·m^2^·s^−1^. Tomato plants were subjected to NB treatments with different frequencies ranging from every 1, 2, 3, and 4 h, and plant growth, flowering, and yield were monitored. The results showed that with the increase of RL NB frequency, plant height decreased, stem diameter increased, and flower initiation delayed, the content of indole-3-acetic acid (IAA) and gibberellin 3 (GA_3_) in the leaf and stem declined. When the RL NB frequency was every 1 h, the heights of tomato plant decreased by 32.73% compared with the control, the diameter of tomato plants increased by 27.09% compared with the control, the number of leaves produced before flowering increased to 11, compared with 8 in the control, the contents of IAA and GA_3_ in the leaf decreased by 33.3 and 41.29% respectively compared with the control, the contents of IAA and GA_3_ in the stem decreased by 56.04 and 57.14% respectively compared with the control. After RL NB treatments, tomato plants were transplanted into a solar greenhouse to evaluate tomato yield. When tomato plants pre-treated with RL NB, per tomato fresh weight of the first spica increased with the increase of RL NB frequencies. These results indicate that more compact and healthier tomato plants could be gotten by RL NB treatments and improve tomato early yield.

## Introduction

The seedling growth period is crucial for vegetable production. This period has important effects on plant growth and development, harvest time, total yield efficiency, and fruit numbers per plant. Seedling optimization can increase production yield and improve the quality of vegetable crops. If plants grow too tall, they become troublesome during propagation, transport, and planting. However, adverse environmental conditions, high humidity, and greenhouse shading often degrade seedling quality (Zhan et al., 2003). To prevent plants from becoming spindly, growers often control seedling height by application of growth retardants such as B-nine (daminozide), Bonzi (paclobutrazol), and Cycocel (chlormequat chloride) (Nourai and Harris, 1983; Berova and Zlatev, 2000; Haque et al., 2007). However, these growth retardants excessively attenuate plant height and slow plant growth after transplanting. Restrictions on the use of growth regulating chemicals have been set on vegetable crops because of potential health risks to workers and consumers. Therefore, identification of a non-chemical alternative to control greenhouse crop height is increasingly important.

Light quality, specifically red light (RL) and far-red light (FRL), affects plant growth. In many morphological studies, high RL:FRL ratio strongly inhibits stem elongation, whereas low RL:FRL ratio enhanced stem elongation (Alokam et al., 2002; Kurepin et al., 2007). Phytochromes are important photoreceptors that sense RL and FRL. Phytochromes are photochromic proteins that have two photo-interconvertible isomeric forms: the red-light-absorbing form (Pr) is biologically inactive, whereas the far-red-light-absorbing form (Pfr) is biologically active (Hughes and Lamparter, 1999; Smith, 2000). The conversion between Pr and Pfr synchronizes plant development with the light environment. In daylight, phytochromes exist predominantly in the Pfr form, which results in the suppression of genes involved in elongation growth. During the night, Pfr undergoes dark recovery and slowly converts into the inactive Pr form, which increases the expression of genes involved in elongation growth (Nozue et al., 2007; Soy et al., 2012). Phytochromes also are involved in flowering, long-day-plants such as *Arabidopsis* (Mockler et al., 1999) and pea (Weller and Reid, 1993), and short-day-plants such as sorghum (Childs et al., 1997) and rice (Izawa et al., 2002), showed early flowering and reduced photoperiodic sensitivities in their *phy* mutants.

A short exposure to light in the middle of the night inhibits or promotes plant growth and flowering. This phenomenon is called night break (NB), which has been used extensively as a tool to study photoperiodic control of growth and flowering. In *Eustoma grandiflorum* and *Cymbidium*, NB treatment by high RL:FRL ratio reduces stem elongation (Yamada et al., 2009; Kim et al., 2011). NB treatment promotes flowering of long-day plants and inhibits flowering of short-day plants (Goto et al., 1991; Ishikawa et al., 2005). NB treatment with RL change phytochromes from the Pr form to the Pfr form during the night. However, the effect of NB treatment on stem elongation and flowering in a day-neutral plant such as tomato was unknown.

Light-emitting diode (LED) is a new light source with several unique advantages, including narrow bandwidth, relatively cool emitting surface, minimum heating, and the ability to control spectral composition and wavelength specificity (Bourget, 2008). These solid-state light sources are therefore ideal for use in plant lighting experiments to influence plant morphology and metabolism (Massa et al., 2008; Morrow, 2008). In this study, we investigated the effects of NB treatment on tomato growth, flowering, and fresh fruit weight using red LED light. The basic knowledge gained by these experiments provides useful information for the development of seedling management practices using specific light manipulation protocols in commercial operations.

## Materials and methods

### Plant materials and treatments

The tomato (*Solanum lycopersicum*) cultivar Jinpeng No.1 was used as experimental material. Tomato seeds were soaked in 50% bleach for 30 min, rinsed thoroughly in running water, and then placed directly on moistened filter paper and incubated at 25°C. After germination, seeds were sown onto culture substrate and placed in a growth chamber with a day/night temperature regime of 25°C/18°C, 12 h photoperiod, and 60–75% relative humidity. When the first leaf fully expanded, the same-sized plants were selected and transplanted to plastic pots (pot size 5 × 5 × 8 cm, one seedling per pot) filled with culture substrate. Then, plants were placed in an environmentally controlled greenhouse at Northwest A&F University, Yangling, Shaanxi, China. The greenhouse was completely enclosed in glass and equipped with pad-and-fan cooling and water heating systems; therefore, the greenhouse could be maintained at a temperature of 25–30°C during the day and 15–18°C during the night, with 60–75% relative humidity. The plants were irrigated every week with Yamasaki nutrient solution (pH 6.5 ± 0.1, electrical conductivity 1.4–1.8 dS·m^−1^) containing 4 mmol·L^−1^ NO_3_-N, 0.7 mmol·L^−1^ NH_4_-N, 0.7 mmol·L^−1^ P, 4 mmol·L^−1^ K, 1.0 mmol·L^−1^ Mg, 1.7 mmol·L^−1^ Ca, 2.7 mmol·L^−1^ S, and micronutrients.

**Figure 1:**
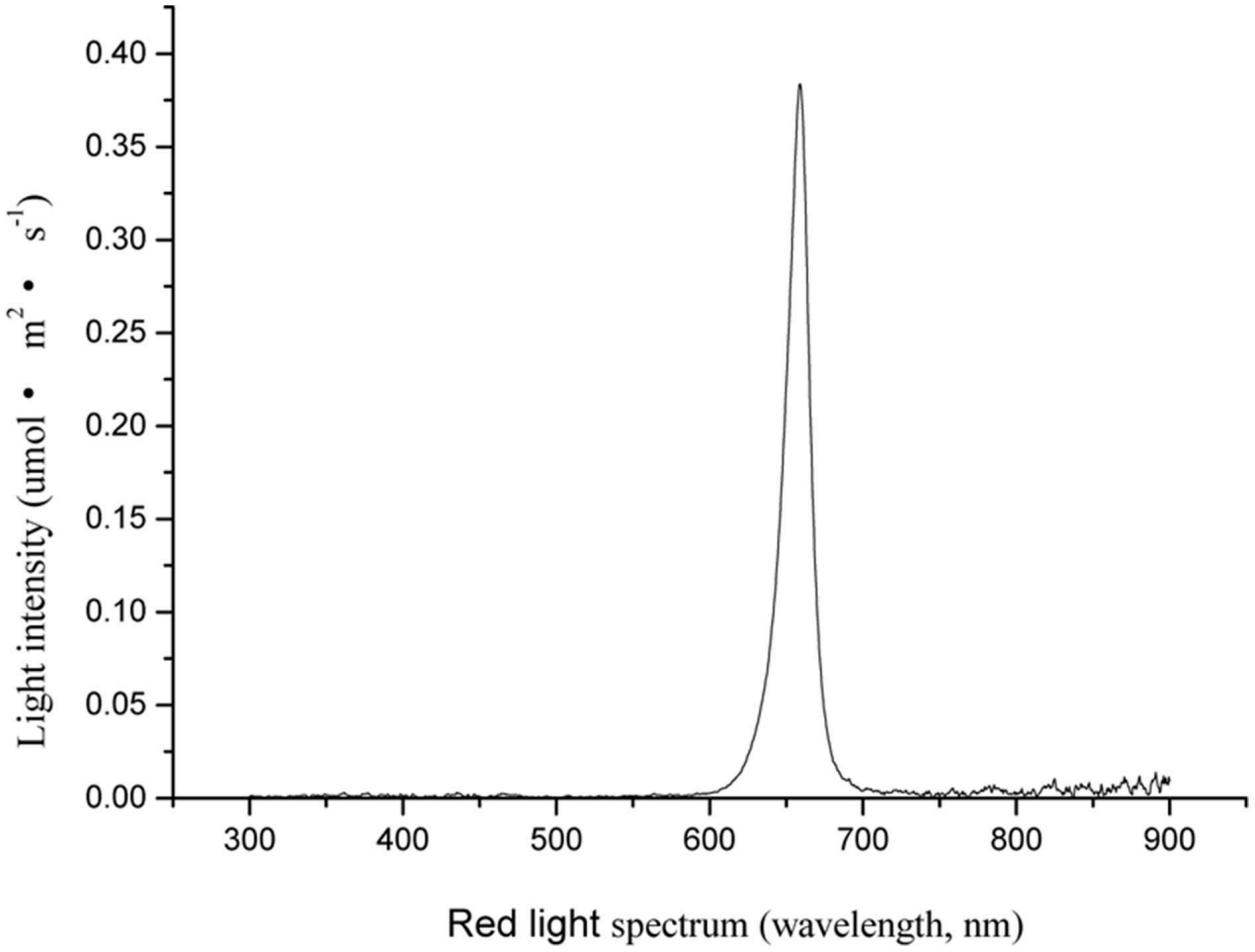
**Spectral distribution characteristics of red LED used for night break (NB) treatment**.

The environmentally controlled greenhouse was divided into five sections. Plants (*n* = 45) were placed in each section and covered with opaque black cardboard box to prevent light contamination. A LED panel (339 × 350 mm, model ISL-RFGB, CCS Inc., Kyoto, Japan) with red LEDs (peak illumination at 658 nm) was horizontally placed 20 cm above the plants inside the opaque cardboard box. The photosynthetic photon flux density of RL was measured by a spectroradiometer (PAR-NIR, Apogee Instruments Inc., Logan, UT). The RL intensity and spectrum are shown in Figure 1. The daily RL NB treatment occurred from 20:00 to 08:00 h, lasting for 10 min at an intensity of 20 μmol·m^2^·s^−1^ inside the opaque cardboard box, with frequencies ranging from every 1, 2, 3, and 4 h. The experiments were conducted in 1–4 black opaque cardboard boxes during the night. The control plants did not receive any NB treatment; they were kept in the fifth black opaque cardboard box during the night, which prevented any light contamination. NB treatments finished when all tomato plants flowered.

### Measurement of plant growth and development during the NB treatment period

Plant height and stem diameter were measured weekly for 10 plants during the NB treatment period. Plant height was measured using a ruler with 0.01 cm accuracy; stem diameter was measured at the first internode using a digital caliper with 0.01 mm accuracy (Digimatic Caliper, Shengli Co., Ltd., Beijing, China). When the plants flowered, 10 plants in each treatment were harvested separately for measurement of leaf, stem, and root dry matter weight (DM). DM was determined after drying tissue at 70°C for 2 days, and then weight was recorded using a balance with 0.01 g accuracy. The time to the start of first inflorescence and the number of leaves below the first inflorescence were recorded.

### Measurement of hormone contents in leaves and stems

To measure the contents of indole-3-acetic acid (IAA) and gibberellin (GA), the third leaf of 3 tomato plants from the top, and the stems of 3 tomato plants were harvested quickly at 08.00 h when RL NB treatments were finished, and were preserved in liquid nitrogen. Approximately 1 g of leaves and stem were extracted with 10 mL of pre-cooled 80% methanol for 24 h at 4°C. The samples were centrifuged at 15,000 × g for 20 min at 4°C, the supernatant was collected, and concentrated into the aqueous phase by placing in a 40°C water bath with rotary evaporator (RE-205, Huayi Instrument Co., Ltd., Shanghai, China). The organic phase was treated with 0.2–0.3 g of polyvinylpolypyrrolidone (PVPP). After another centrifugation at 15,000 × g for 20 min at 4°C, the supernatant was adjusted to pH 2.5–3.0, extracted three times with an equal volume of ethyl acetate, and finally evaporated to dryness with a rotary evaporator at 40°C as described above.

The dried samples containing IAA and GA_3_ were dissolved in chromatography grade methanol with 0.1 M glacial acetic acid as the mobile phase. The flow rate for all analyses was adjusted to 1 mL/min. Samples (100 μL) were subjected to high performance liquid chromatography (HPLC) analysis (Dobrev and Vankova, 2012; Yang et al., 2014). Detection and analysis were performed using a Waters 2498/UV Visible Detector (Shaanxi, China) (Djilianov et al., 2013). The analysis of IAA and GA_3_ contents were repeated with 3 biological replicates, and each sample was assayed in triplicate by HPLC.

### Measurement of plant growth, development, and yield after NB treatment

NB treatments were finished when all plants began flowering. Then, tomato plants were transplanted into a solar greenhouse at the Experimental Station of Horticulture Department, Northwest A&F University P.R. China. The greenhouse is 76 m long and 8 m wide, with a planting area of 405 m^2^. Construction of the solar greenhouse was described by Qiu et al. (2011). For each NB treatment group, 30 tomato plants were evenly transplanted along the edge of the furrow side with row spacing of 0.35 m and interplant spacing of 0.35 m. Plant height, stem diameter, and dry mass were determined at 2, 5, and 8 weeks after 10 tomato plants were transplanted into the solar greenhouse. Fresh fruit yield per plant and individual fruit weights were measured for each plant of 10 plants measured at harvest.

### Statistical analysis

Data were analyzed with ANOVA and Duncan's multiple range tests using the SAS software package (version 8.0, SAS Institute Cary, NC, USA). Graphing was performed in Excel 2007 or OriginPro (version 8.0, Origin Lab, MA, USA).

## Results

### Effects of RL NB treatments on tomato vegetative growth

**Figure 2:**
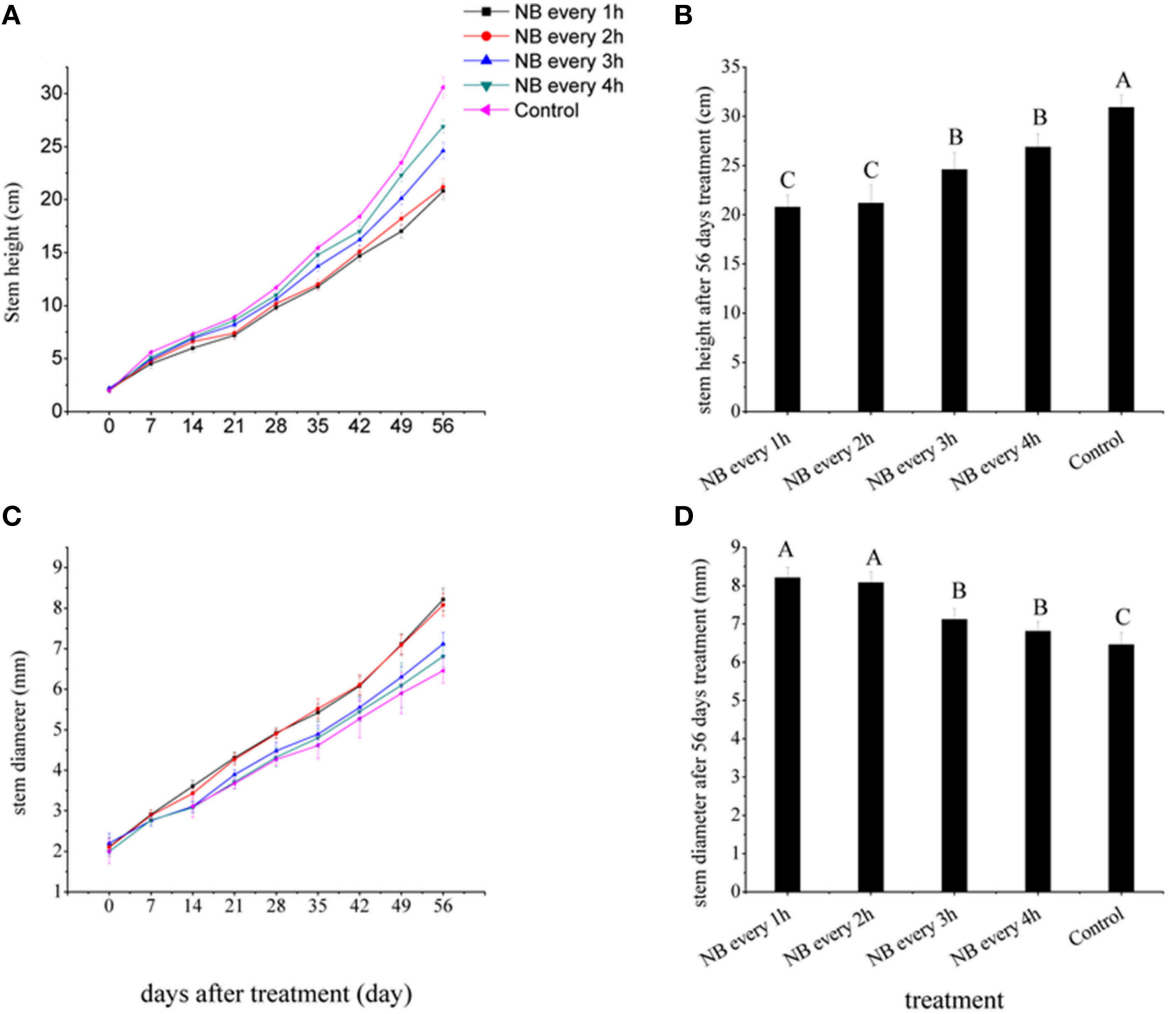
**Effects of red light (RL) night break (NB) treatment on tomato plant height and stem diameter. (A)** Plant height was measured weekly after RL NB treatments every 1, 2, 3, and 4 h. **(B)** Plant height was measured after 56 days of RL NB treatments every 1, 2, 3, and 4 h. **(C)** Stem diameter was measured weekly after RL NB treatments every 1, 2, 3, and 4 h. **(D)** Stem diameter after 56 days of RL NB treatments every 1, 2, 3, and 4 h. Vertical bars represent SE (*n* = 10). Bars with different letters are significantly different at the level of *P* = 0.01 (Duncan's multiple range test).

To study the effect of NB treatments on tomato seedling stem elongation, we first determined the RL NB treatments that were most effective to inhibit stem elongation in tomato plants. When the first leaf expanded, The RL NB treatments were applied to tomato plants when the first leaf expanded, were scheduled to last for 10 min, and recurred with a frequency of 1, 2, 3, and 4 h from 20:00 to 08:00 h. Figure 2A shows that all RL NB treatments inhibited stem elongation of tomato plants. The RL-treated plants were shorter than the control plants. After 56 days of RL NB treatments, the heights of tomato plants exposed to RL NB every 1, 2, 3, and 4 h decreased by 32.73, 31.44, 20.44, and 13.00%, respectively, compared with those of the control plants (Figure 2B).

The stem diameters of tomato plants were strongly affected by RL NB treatments (Figure 2C). After 56 days of treatments, the stem diameters of tomato plants subjected to RL NB every 1, 2, 3, and 4 h increased by 27.09, 25.08, 10.22, and 6.07%, respectively, compared with those of control plants (Figure 2D). No differences in total dry weight of tomato plants were observed between the RL NB treatment groups and the control group (Table 1). However, the leaf dry weight significantly increased and the stem dry weight significantly decreased in plants subjected to RL NB treatment every 1 and 2 h compared with those of the control plants (Table 1).

**Table 1:** **Effect of red light night break (NB) on the dry weight of tomato leaf, stem, and root**.

**Treatment**	**Leaf dry weight (g)**	**Stem dry weight (g)**	**Root dry weight (g)**	**Total dry weight (g)**
NB every 1 h	0.86^A^	0.37^B^	0.15^A^	1.39^A^
NB every 2 h	0.84^A^	0.38^B^	0.14^A^	1.36^A^
NB every 3 h	0.77^B^	0.44^AB^	0.13^A^	1.34^A^
NB every 4 h	0.75^B^	0.48^A^	0.13^A^	1.36^A^
Control	0.72^*B*^	0.53^A^	0.12^A^	1.37^A^

*Different letters in a column represent significance at the 0.01 level (Duncan's multiple range test)*.

### Effects of RL NB treatments on flowering of tomato plants

**Figure 3:**
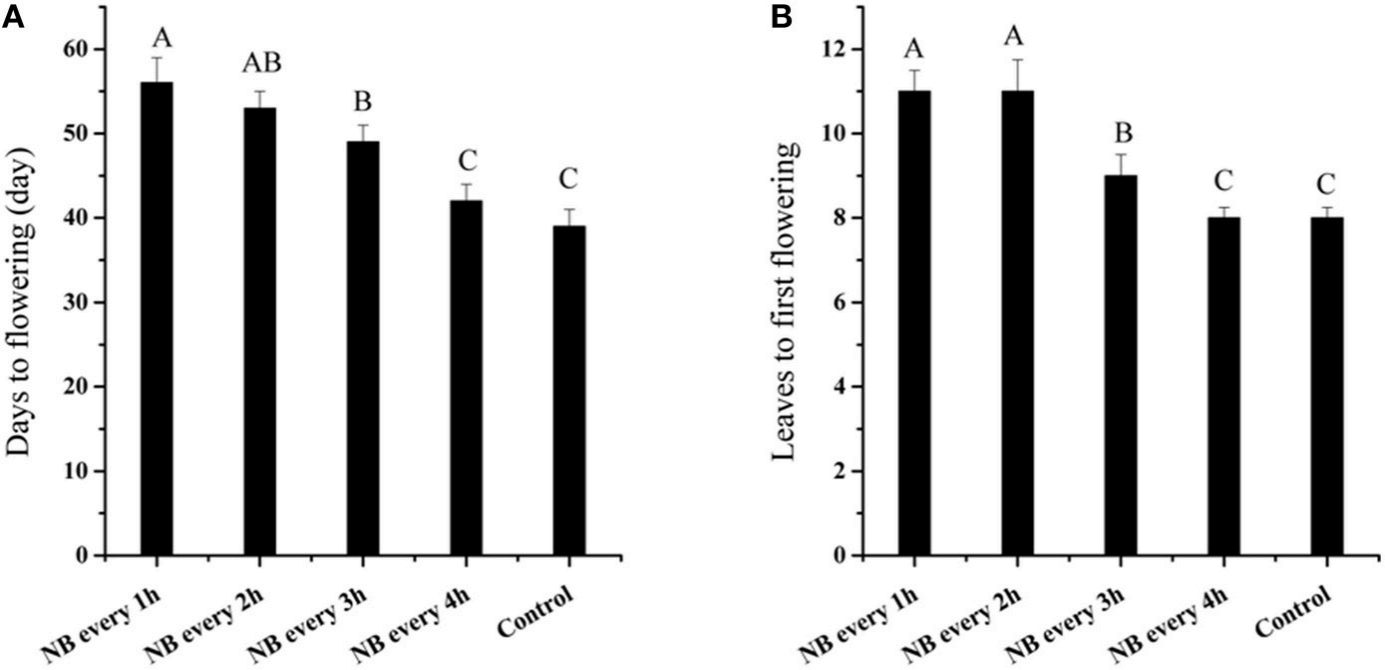
**Effects of red light (RL) night break (NB) on flowering of tomato plants. (A)** Effects of RL NB treatment every 1, 2, 3, and 4 h on the number of days to flowering. **(B)** Effects of RL NB treatment every 1, 2, 3, and 4 h on the number of leaves to flowering. Vertical bars represent SE (*n* = 3). Bars with different letters are significantly different at the level of *P* = 0.01 (Duncan's multiple range test).

The transition from vegetative growth to reproductive growth is a major event in the plant cycle. The RL NB treatments delayed the flowering time of tomato plants. Control plants produced 8 leaves on the main stem before flowering; however, plants treated with RL NB every 1 and 2 h produced 11 leaves before flowering (Figure 3A), and those treated with RL NB every 3 h produced an average of 9 leaves before flowering (Figure 3A). There were no differences in the number of leaves produced before flowering in plants subjected to RL NB treatment every 4 h and control plants. The time from the start of the experiment to flowering was 39 days for control plants. The time to flowering for tomato plants subjected to RL NB treatment every 1, 2, 3, and 4 h increased to 56, 53, 49, and 42 days, respectively (Figure 3B).

### Effects of RL NB treatments on IAA and GA_3_ contents

**Figure 4:**
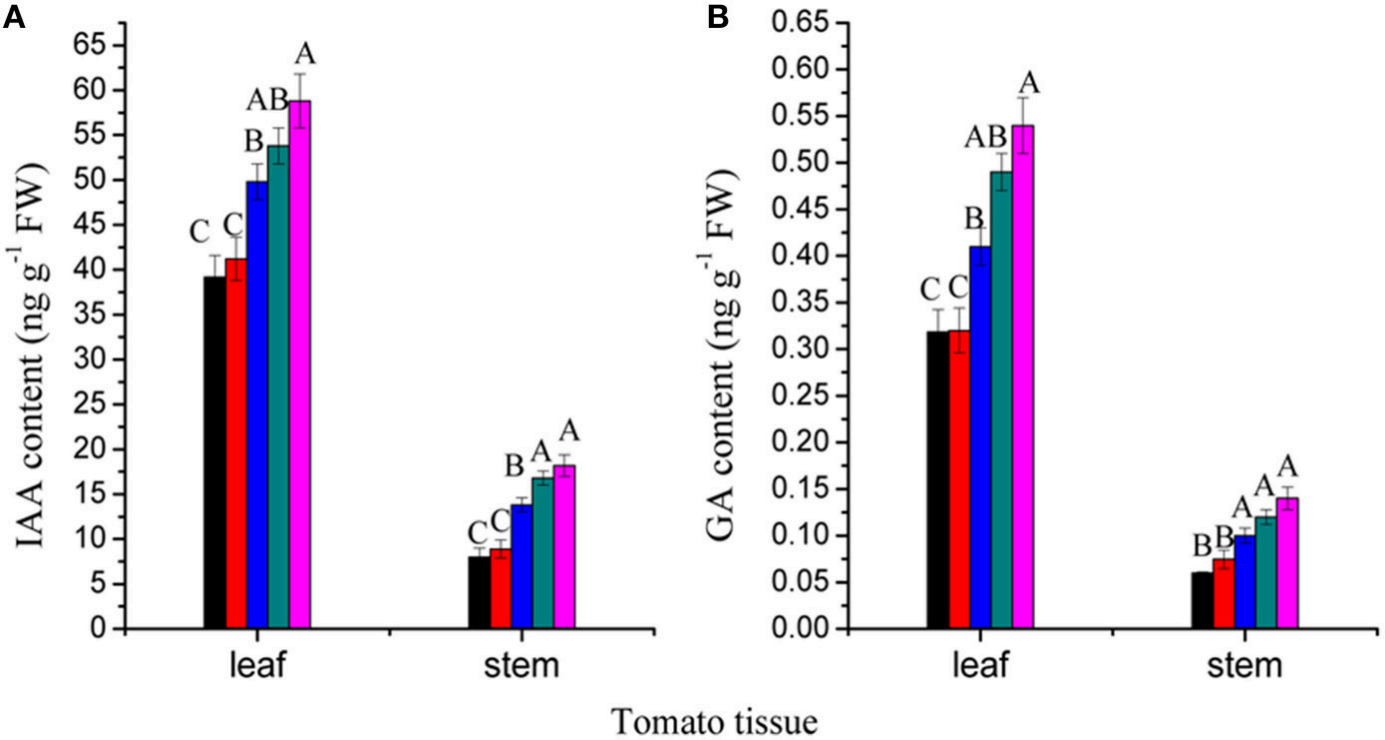
**Effects of red light (RL) night break (NB) on phytohormone contents in leaves and stems of tomato plants. (A)** Effects of RL NB treatment every 1, 2, 3, and 4 h on IAA contents in tomato leaves and stems. **(B)** Effects of RL NB treatment every 1, 2, 3, and 4 h on GA contents in tomato leaves and stems. Vertical bars represent SE (*n* = 3). Bars with different letters are significantly different at the level of *P* = 0.01 (Duncan's multiple range test).

IAA and GA have important roles in plant growth and development. We measured the contents of IAA and GA_3_ in leaves and stems of tomato plants subjected to RL NB treatment for 56 days. The results showed that IAA and GA_3_ contents in both leaves and stems decreased after RL NB treatment (Figures 4A,B). Treatment of tomato plants with RL NB every 1, 2, 3, and 4 h reduced the IAA contents in leaves by 33.3, 29.93, 15.31, and 8.50%, respectively, and reduced the GA_3_ content in leaves by 41.29, 40.74, 24.07, and 9.26%, respectively, compared with those of control plants (Figures 4A,B). The RL NB treatment every 1, 2, 3, and 4 h reduced the IAA and GA_3_ contents in stem by 56.04, 51.09, 24.17, and 7.69%, respectively, and 57.14, 46.71, 28.57, and 14.28%, respectively, compared with those of control plants (Figures 4A,B).

### Effects of RL NB treatments on mature tomato plant growth and fresh fruit weight

The quality of plants and young plants is important for plant growth and yield. Two weeks after the young tomato plants were transplanted into the solar greenhouse, the stem diameter and leaf dry mass of tomato plants treated with RL NB every 1 and 2 h increased significantly, whereas plant height and stem dry weight decreased, and root dry weight did not change compared with control plants (Table 2). No significant differences in plant height, stem diameter, leaf dry mass, and stem dry mass were detected 5 weeks after tomato plants were transplanted into the solar greenhouse.

**Table 2:** **Growth and development of tomato plants in the solar greenhouse after red light night break (NB) treatment of plants and young plants up until flowering**.

**Treatment**	**2 weeks in solar greenhouse**					**5 weeks in solar greenhouse**					**8 weeks in solar greenhouse**				
	**Plant height (cm)**	**Stem diameter (cm)**	**Dry mass (g)**	**Plant height (cm)**	**Stem diameter (cm)**	**Dry mass (g)**	**Plant height (cm)**	**Stem diameter (cm)**	**Dry mass (g)**
			**Leaf**	**Stem**	**Root**			**Leaf**	**Stem**	**Root**			**Leaf**	**Stem**	**Root**
NB every 1 h	48.12^B^	11.96^A^	6.18^A^	2.36^A^	1.36^A^	92.12^A^	14.58^A^	18.66^A^	8.34^A^	2.09^A^	157.82^A^	16.21^A^	34.81^A^	14.88^A^	4.44^A^
NB every 2 h	49.02^B^	10.72^A^	6.07^A^	2.22^A^	1.32^A^	92.19^A^	14.45^A^	18.52^A^	7.95^A^	1.99^A^	155.71^A^	15.99^A^	33.78^A^	15.03^A^	4.71^A^
NB every 3 h	54.42^AB^	9.77^B^	5.34^B^	2.18^AB^	1.18^A^	90.18^A^	13.18^A^	16.93^A^	7.92^A^	2.11^A^	157.23^A^	15.38^A^	30.77^A^	14.92^A^	4.55^A^
NB every 4 h	55.58^A^	8.55^C^	4.85^C^	2.11^B^	1.26^A^	90.14^A^	13.08^A^	17.18^A^	7.83^A^	1.98^A^	153.35^A^	15.62^A^	33.34^A^	14.55^A^	4.18^A^
Control	57.85^A^	8.08^C^	4.46^C^	1.99^B^	1.10^A^	89.95^A^	12.99^A^	17.11^A^	7.99^A^	2.06^A^	150.59^A^	15.41^A^	33.41^A^	13.72^A^	4.62^A^

*Different letters in a column represent significance at the 0.01 level (Duncan's multiple range test)*.

After the tomato plants pre-treated with RL NB treatment every 1, 2, 3, and 4 h, the per tomato fresh weight increased by 23.58, 18.39, 12.26, and 9.43%, respectively, compared with control plants (Table 3). The increased fresh weight per tomato plant is due to the increased fresh weight of the first spica. There were no differences in the fresh weights of the second and third spica for plants subjected to RL NB treatments and control plants (Table 3).

**Table 3:** **Effect of red light night break (NB) on tomato growth, development, and fruit yield**.

**Treatment**	**Per fruit mass of first spica (g)**	**Per fruit mass of second spica (g)**	**Per fruit mass of third spica (g)**	**Fresh fruit mass per plant (kg)**
NB every 1 h	215.561^A^	211.232^A^	204.522^A^	2.62^A^
NB every 2 h	207.348^A^	219.311^A^	198.451^A^	2.51^A^
NB every 3 h	178.145^B^	207.431^A^	211.072^A^	2.38^AB^
NB every 4 h	146.235^B^	207.174^A^	209.812^A^	2.23^B^
Control	138.036^C^	203.321^A^	216.216^A^	2.12^B^

*Different letters in a column represent significance at the 0.01 level (Duncan's multiple range test)*.

## Discussion

Previous studies on stem elongation kinetics indicated that stem elongation was not constant during a 24 h day/night cycle, in many plant species, stem elongation rate was higher in dark conditions than in the light (Bertram and Karlsen, 1994; Tutty et al., 1994). Therefore, one of the effective ways to control plant height is to control the light condition in the dark period. Recent studies report that RL or high R: Fr light can make phytochromes exist more in Pfr form which inhibit plant stem elongation, whereas FRL or low R: Fr light can make phytochromes exist more in Pr form which can promote plant stem elongation (Behringer and Davies, 1992; Van Tuinen et al., 1999; Smith, 2000). Tomato contains five phytochromes, designated PHYA, PHYB1, PHYB2, PHYE, and PHYF, the five phytochromes can sense RL and FRL to control tomato plant growth (Hauser et al., 1997). It has been concluded that PHYB1 is mainly responsible for mediating the de-etiolation response of seedlings to RL as quantified by the inhibition of hypcotyl elongation, enhancement of anthocyanin accumulation, and end-of-day FRL responses (Kerckhoffs et al., 1997). Tomato plants subjected to light environments with low R:FR ratio or end-of-day FRL treatment displayed no differences in leaf area expansion, but had significantly increased stem elongation (Chia and Kubota, 2010; Kurepin et al., 2010). In the current study, we use RL NB treatment to prevent tomato plants from becoming spindly. RL NB can quickly change phytochromes from the Pr form to the Pfr form during the night and inhibit tomato stem elongation. The results showed that with the increase of RL NB frequency, plant height decreased. When the RL NB frequency was every 1 h, the heights of tomato plant decreased by 32.73% compared with the control (Figures 2A,B).

Before entering dark conditions, phytochromes exist predominantly in the Pfr form. After entering dark conditions, Pfr undergoes dark recovery and slowly converts into the inactive Pr form. 10 min RL NB can quickly change phytochromes from the Pr form to the Pfr form. When RL NB frequency is every 4 h, the tomato plants will enter dark period 4 h after 10 min RL NB which will lead phytochromes undergoes dark recovery and slowly converts into the inactive Pr form during the 4 h dark period. Therefore, the inhibition of tomato stem elongation by RL NB every 4 h is limited. However, when RL NB frequencies is every 1 h, the tomato plants will enter dark period 1 h after 10 min RL NB which will not enough for dark recovery. Therefore, the inhibition of tomato stem elongation by RL NB every 1 h is significant.

Light and phytohormones did not always independently regulate plant growth. This study showed that IAA and GA_3_ contents decreased in both leaf and stem after treatment with RL NB, which reduced tomato stem elongation. Similar results have been reported by Steindler et al. (1999), who observed that inhibition of auxin transport alone was sufficient to abolish hypocotyl elongation in *Arabidopsis* plants grown under low R:FR ratio. Behringer and Davies (1992) reported that end-of-day FRL treatment increased IAA levels and stem elongation in the third internode of *Pisum sativum* plants. Kurepin et al. (2010) reported that stem elongation and endogenous GA levels increased when tomato plants were grown under low R:FR ratio conditions. Van Tuinen et al. (1999) demonstrated that tomato plants subjected to end-of-day FRL treatment elongated significantly more than control plants which could be due to the fact that phytochromes exist more in the Pr form during the night after end-of-day FRL treatment. In this study, RL NB treatment caused rapid conversion of the phytochromes from the Pr form to the Pfr form, which reduced the expression of genes involved in hormone synthesis, decreased the content of IAA and GA_3_ in tomato leaf and stem, and inhibited tomato stem elongation (Nozue et al., 2007; Soy et al., 2012).

Tomato is a photoperiod-insensitive plant. The primary shoot is terminated by an inflorescence, and subsequent upright growth of tomato plants manifests as an apparent linear shoot consisting of consecutive sympodial units, each producing three leaves before terminating in a compound inflorescence. Tomato flowering time was evaluated by the number of leaves produced in the initial segment. Flower initiation was earlier and inflorescence development was superior under short-day conditions than under long-day conditions (Kinet, 1977). The NB treatments affected flowering of both long-day (Goto et al., 1991) and short-day plants (Ishikawa et al., 2005), but the effects were more evident in short-day plants, in which flowering was inhibited by a very short exposure to light during the night (Ishikawa et al., 2005). In the present study, the number of leaves to the first inflorescence increased from 8 in the control to 11 after tomato plants suffer from RL NB treatment every 1 and 2 h, but the number of inflorescences and florets did not differ from those of control plants. The sympodial unit morphologies of tomato plants subjected to RL NB treatments were the same as those of control plants, with each producing three leaves before terminating in a compound inflorescence (data not shown). Daily exposure to 1 h of light in the middle of the night resulted in early flowering in *Arabidopsis* (Goto et al., 1991). Treatment of rice (*Oryza sativa*) with RL NB for 10 min clearly affected flowering when applied for different numbers of days (Ishikawa et al., 2005). In *Eustoma grandiflorum*, NB treatment using light with R:FR ratio above 5.3 delayed flowering, whereas light with R:FR ratio below 5.3 promoted earlier flowering (Yamada et al., 2009; Kim et al., 2011). In the future tomato growth, we can control the number of leaves to flowering through RL NB treatments.

Tomato is widely grown worldwide, and its production is economically and culturally important. The production of compact and heathier tomato plants could ultimately increase plant yield. This study showed that the fresh weight of the first spica increased after RL NB treatment. This resulted from an increase in the number of leaves from 8 to 11 in response to RL NB treatment every 1 and 2 h. The model developed by Heuvelink (1996) proposes that dry matter distribution in fruiting vegetable crops is regulated primarily by organ sink strength. Fruit development is an important event that significantly changes the sink load. From the time of inception, tomato fruit may account for as much as 90% of the total increase in plant dry weight (Nielsen and Veierskov, 1988). The RL NB treatment delayed flowering time in tomato plants, and plants produced more leaves, which increased leaf dry matter distribution, enhanced photosynthesis, and accelerated plant growth. This resulted in higher fresh weight of the first spica after treatment of tomato plants with RL NB.

LED is a new light source with several unique advantages, the use of LED in protected agriculture will become more and more popular. Therefore, the research of the precision utilization of LED in protected horticulture is very important for energy-saving and accuracy control in protected vegetable growth. Based on the results we obtained in this study, R LED could be used as a non-chemical alternative to control tomato height. More compact and healthier tomato plants could be gotten by NB using R LED and improve tomato early yield.

## Author contributions

ZZ conceived and designed research; KC, LC, LY, XZ, and HZ conducted the experiments and write this manuscript, EB revised the language.

### Conflict of interest statement

The authors declare that the research was conducted in the absence of any commercial or financial relationships that could be construed as a potential conflict of interest.
